# Recurrent Chondromyxoid Fibroma of the Second Toe Distal Phalanx Treated with Distal Phalangectomy: A Case Report

**DOI:** 10.3390/children13040552

**Published:** 2026-04-16

**Authors:** Jun Yong Park, Woo-Jong Kim, Dong Woo Lee, Byungsung Kim, Hyun Deuk Cho, Ki Jin Jung

**Affiliations:** 1 Department of Orthopaedic Surgery, Soonchunhyang University Cheonan Hospital, 31 Sooncheonhyang 6-gil, Dongnam-gu, Cheonan 31151, Republic of Korea; 142146@schmc.ac.kr (J.Y.P.); kwj9383@hanmail.net (W.-J.K.); 122923@schmc.ac.kr (D.W.L.); 2 Department of Orthopaedic Surgery, Soonchunhyang University Hospital Bucheon, 170, Jomaru-ro, Bucheon-si 14584, Republic of Korea; kbsos@schmc.ac.kr; 3 Department of Pathology, Soonchunhyang University Cheonan Hospital, 31 Sooncheonhyang 6-gil, Dongnam-gu, Cheonan 31151, Republic of Korea; path95@schmc.ac.kr

**Keywords:** bone neoplasms, chondromas, toe phalanges, neoplasm recurrence, local, amputation, surgical

## Abstract

**Background/Objectives**: Chondromyxoid fibroma (CMF) is a rare benign cartilaginous bone tumor, accounting for less than 1% of all primary bone tumors. Although CMF most commonly arises in the metaphysis of long bones, involvement of the phalanges of the toes is uncommon. We report a pediatric case of CMF arising in the distal phalanx of the second toe that recurred four years after initial surgical treatment and discuss its management. **Methods**: A 10-year-old girl presented with a painless mass in the distal phalanx of the second toe. Imaging studies demonstrated an expansile osteolytic lesion with cortical thinning, showing a somewhat aggressive radiologic appearance. Intralesional curettage and debridement were performed to preserve the digit, and the bone defect was reconstructed using allogenic cancellous bone graft and demineralized bone matrix. Histopathological examination confirmed the diagnosis of CMF. **Results**: Four years later, the patient returned with progressive enlargement of the lesion, indicating tumor recurrence. Because of the recurrent nature of the tumor and progressive cosmetic deformity and recurrence-related anxiety, distal phalangectomy was performed. At the one-year follow-up, the patient showed no evidence of recurrence and maintained satisfactory functional and cosmetic outcomes. **Conclusions**: CMF of the toe phalanx may show delayed recurrence after curettage, requiring prolonged radiologic surveillance. In recurrent cases, definitive resection should be considered based on a combination of oncologic, anatomic, and patient-centered factors, with distal phalangectomy providing reliable local control in anatomically expendable digits.

## 1. Introduction

Chondromyxoid fibroma (CMF), first described by Jaffe and Lichtenstein in 1948 [1], is a rare benign cartilaginous bone tumor, accounting for less than 1% of all primary bone tumors and approximately 2% of benign bone tumors [2,3]. CMF most commonly arises in the metaphysis of long bones, particularly the proximal tibia and distal femur, and predominantly affects adolescents and young adults [2,4]. Although CMF is considered a benign neoplasm, it is known for its potential for local recurrence, particularly following intralesional curettage [3,5,6].

Because of its rarity in this anatomical location, CMF involving the toe phalanx may present diagnostic and therapeutic challenges and can mimic other benign or low-grade cartilaginous lesions such as enchondroma or low-grade chondrosarcoma [2,3,7,8].

We report a pediatric case of CMF arising in the distal phalanx of the second toe—an anatomical site not previously described in the literature—that recurred four years after initial curettage. This case highlights the potential for late recurrence and discusses the therapeutic considerations—including oncologic control, anatomic expendability, and patient-centered factors such as cosmetic concern and recurrence-related anxiety—when managing CMF in anatomically constrained digits.

## 2. Case Report

A 10-year-old girl presented with a painless, slowly enlarging mass on the left second toe that had been noticed several months prior to presentation. There was no history of trauma or infection, no family history of bone tumors or musculoskeletal neoplasms, and the patient had no significant past medical history. Physical examination revealed a firm, non-tender mass localized to the distal phalanx of the second toe without overlying skin changes or nail deformity.

**Figure 1:**
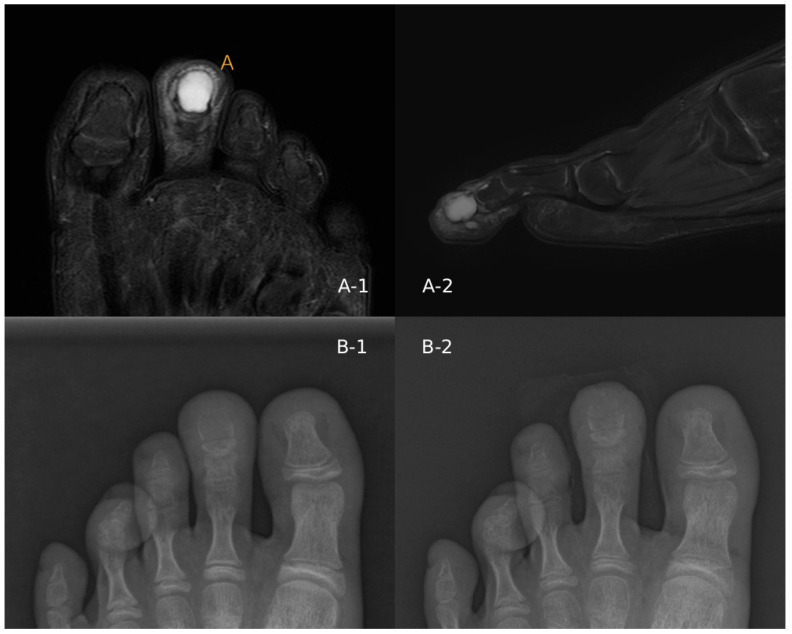
Preoperative imaging findings. (**A-1**) Axial FS-T2 MR: expansile lesion confined to the distal phalanx. (**A-2**) Sagittal FS-T2 MR: lesion extent. (**B-1**) Plain radiograph: osteolytic lesion with cortical expansion, no cortical destruction. (**B-2**) Postoperative radiograph after curettage and bone grafting.

Initial radiographic evaluation demonstrated a well-defined expansile osteolytic lesion confined to the distal phalanx. Magnetic resonance imaging revealed a localized lesion with high signal intensity on fat-suppressed T2-weighted images. The lesion showed mild cortical expansion and thinning, giving a somewhat aggressive radiologic appearance. However, considering the patient’s age and the lesion location in a small tubular bone, benign cartilaginous tumors such as enchondroma were considered the most likely diagnosis. Because chondromyxoid fibroma is extremely rare in the toe phalanx, it was not strongly suspected preoperatively (Figure 1).

Given the diagnostic uncertainty and the importance of digit preservation in a growing child, intralesional curettage was selected as the initial approach to allow for simultaneous histopathological confirmation and treatment. An excisional biopsy with intralesional curettage and debridement was therefore performed through a cortical window to preserve the digit and minimize functional impairment. The lesion was thoroughly curetted and the cavity was irrigated. The resulting bone defect was filled with allogenic cancellous bone graft combined with demineralized bone matrix (DBM).

**Figure 2:**
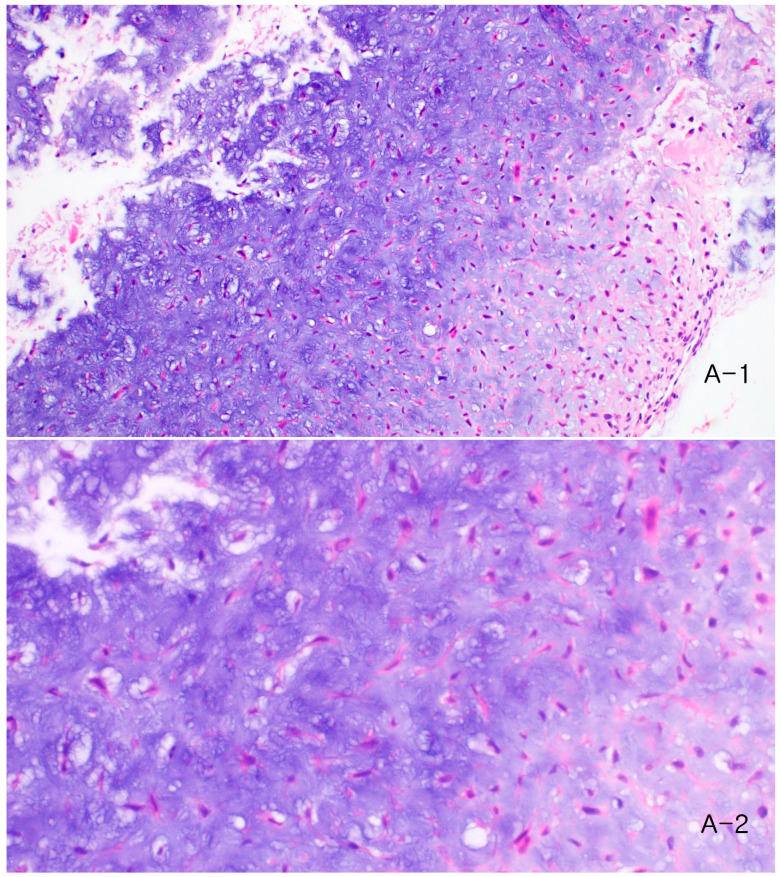
Histopathologic findings from the initial surgery. (**A-1**) Low-power view (100×) showing lobulated chondroid areas. (**A-2**) High-power view (400×) demonstrating chondroid tissue with cartilage-like cells and myxoid stroma containing stellate cells.

Histopathological examination demonstrated lobulated areas composed of chondroid and myxoid stroma with stellate or spindle-shaped cells, consistent with chondromyxoid fibroma (Figure 2). Immunohistochemical staining showed focal smooth muscle actin (SMA) positivity, while S-100 protein and CD34 were negative. The postoperative course was uneventful.

The patient was followed clinically and radiographically during the early postoperative period and remained asymptomatic. However, the patient was subsequently lost to follow-up for approximately three years, returning at age 14 with symptoms of recurrence.

**Figure 3:**
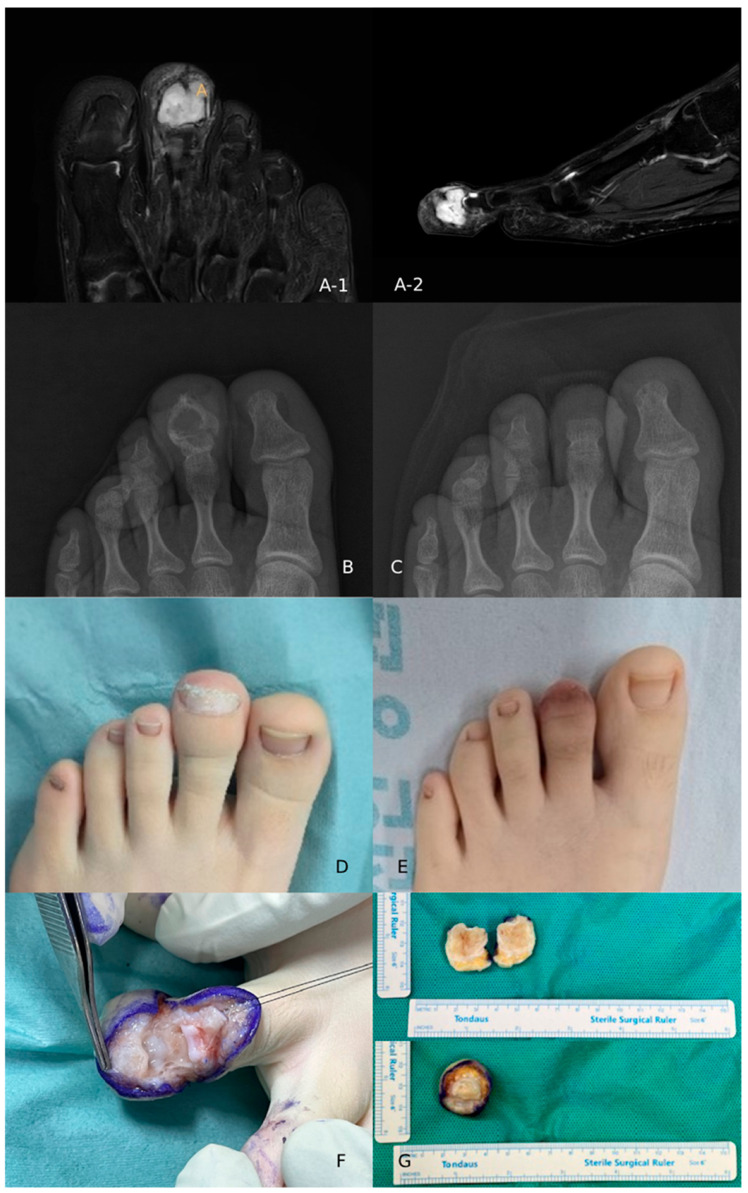
Imaging findings at recurrence. (**A-1**) Axial FS-T2 MR: enlarged hyperintense lesion occupying the distal phalanx. (**A-2**) Sagittal FS-T2 MR: cortical thinning and focal cortical breach. (**B**) Preoperative radiograph: recurrent expansile osteolytic lesion. (**C**) Postoperative radiograph after distal phalangectomy. (**D**) Preoperative clinical photograph: swelling of the second toe. (**E**) Postoperative clinical photograph: cosmetic outcome. (**F**) Intraoperative photograph: surgical field with ink-marked margins and exposed lobulated tumor. (**G**) Gross specimen (cross-sectional and longitudinal views): lobulated yellowish-white tumor filling the medullary cavity.

Four years later, at the age of 14, the patient returned with progressive enlargement of the mass at the same site. Repeat radiographs and magnetic resonance imaging demonstrated a recurrent expansile lesion involving the distal phalanx with cortical thinning and focal cortical breach, suggesting locally aggressive behavior (Figure 3).

Although the patient did not report significant pain or functional limitation, the lesion had caused visible enlargement and deformity of the toe, leading to significant cosmetic distress. Additionally, the patient and her family expressed considerable anxiety regarding the recurrent nature of the tumor and the uncertainty of further disease progression. Given the recurrent nature of the tumor, the documented focal cortical breach on imaging, and the limited surgical margin within the distal phalanx, repeat curettage was considered unlikely to provide durable local control.

**Figure 4:**
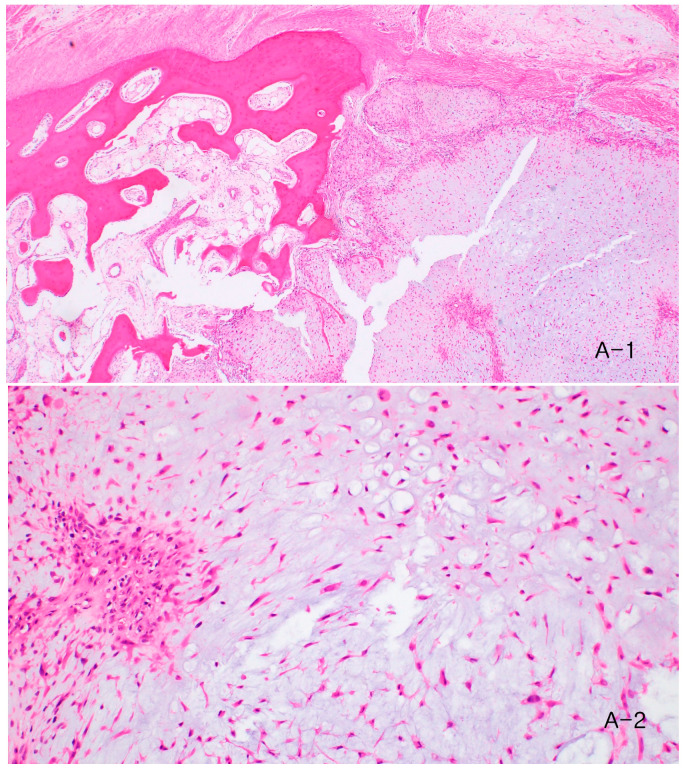
Histopathologic findings of the recurrent lesion. (**A-1**) Low-power view (40×) showing lobulated chondroid and myxoid components. (**A-2**) High-power view (200×) demonstrating cartilage-like cells and stellate cells within the myxoid matrix.

After discussion with the patient and her family, distal phalangectomy of the second toe was performed to achieve complete tumor excision and to address the progressive deformity. Histopathological analysis of the resected specimen again confirmed the diagnosis of chondromyxoid fibroma (Figure 4). The postoperative course was uneventful. At the most recent follow-up, one year after surgery, the patient reported high cosmetic satisfaction and relief from recurrence-related anxiety, and demonstrated a normal gait without pain or functional impairment, and no evidence of recurrence has been observed. The total observation period from the initial surgery to the most recent follow-up is five years.

This case was managed at a university-affiliated hospital with a dedicated multidisciplinary bone tumor management team, comprising orthopedic surgery, diagnostic radiology, medical oncology, radiation oncology, and pathology. Regular tumor board discussions were conducted at both the initial and recurrent presentations.

## 3. Discussion

CMF is a rare benign cartilaginous neoplasm, accounting for less than 1% of all primary bone tumors and approximately 2% of benign bone tumors [3,5]. It most commonly occurs in the metaphysis of long bones—particularly the proximal tibia and distal femur—while involvement of the short tubular bones of the hands and feet is distinctly uncommon [3,9]. Toe involvement represents fewer than 5% of reported cases [3,9], and distal phalangeal lesions are exceedingly uncommon. Previous reports have documented only a limited number of CMF cases in the great toe distal phalanx [8,9,10]; thus, CMF in the second toe distal phalanx of a pediatric patient represents an unusual anatomical and demographic presentation.

Clinically, CMF typically presents with localized pain and swelling. In the present case, however, the patient reported no pain at either initial presentation or recurrence—a finding that highlights the variable clinical spectrum of CMF and underscores the importance of recognizing cosmetic and psychological burden as clinically valid concerns when functional symptoms are absent. On plain radiographs, CMF appears as a well-demarcated, eccentric osteolytic lesion with a sclerotic rim and no periosteal reaction, contrasting with low-grade chondrosarcoma, which may demonstrate endosteal scalloping, cortical erosion, or ring-and-arc matrix mineralization [7,8,9]. On MRI, CMF characteristically demonstrates high signal intensity on fat-suppressed T2-weighted sequences, with a relatively low T1 signal and a lobulated outline; periosteal reaction and soft tissue extension are typically absent, further distinguishing it from chondrosarcoma [4,7,11]. In the present case, the initial MRI showed a T2-hyperintense lesion confined to the distal phalanx without cortical breach, consistent with a benign process and supporting the initial conservative management decision. At recurrence, focal cortical breach was confirmed on both MRI and plain radiograph, indicating locally more aggressive behavior; this directly supported the decision for definitive resection over repeat curettage.

Histopathological evaluation remains the gold standard for diagnosis. CMF has a lobulated architecture with chondroid and myxoid areas containing stellate or spindle-shaped cells [7,8,9]. CMF may demonstrate focal cellular atypia that can raise concern for malignancy; however, such features represent “pseudo-malignant” degenerative change rather than true malignant transformation [8]. The key pathological distinguishing feature from chondrosarcoma is the zonation pattern—peripheral hypercellularity with central hypocellularity—which is absent in chondrosarcoma [5]. Atypical mitotic figures are characteristically absent in CMF; their presence should prompt reclassification [5]. In diagnostically challenging cases, GRM1 immunohistochemistry may serve as a useful ancillary marker, with reported positivity in 97% of CMF specimens and negativity in all histologic mimics [12]; this was not performed in the present case, as the marker was not yet in routine clinical use at the time of diagnosis.

In the present case, intralesional curettage with bone grafting was the appropriate initial approach for a primary lesion in a growing child with no preoperative suspicion of CMF—consistent with the management reported by Vasudeva et al. [8] for a similar pediatric case. Recurrence four years later, despite the use of allogenic cancellous bone graft combined with demineralized bone matrix, is consistent with published evidence that bone grafting reduces but does not eliminate local recurrence in CMF [6], and that delayed recurrence may occur even when bone grafting is performed [5,13]. Mallya and Sujir [13] reported recurrence six years after curettage and fibular bone grafting, ultimately requiring amputation. These observations reflect the inherent challenge of achieving durable local control in small tubular bones—not a failure of the initial surgical strategy. Previously reported cases of CMF involving the digital phalanges are summarized in Table 1.

At recurrence, standard limb-sparing adjuvants—phenol cauterization, polymethylmethacrylate (PMMA) cement augmentation, cryotherapy, and extended curettage—were considered and rejected on anatomic grounds: the cortical walls were too thin for safe cryoprobe use; phenol carries significant risk of nail bed injury in this location; and cement augmentation offers little structural benefit in such a confined space. HemanthaKumar and Sathish [2] found that phenol and liquid nitrogen did not reduce recurrence in their CMF series. Mechanical adjuvant (electrocauterization and burring) achieved a 3.7% recurrence rate in Karaca et al.’s series [4], but only where cortical margins were intact—a condition precluded here by the confirmed cortical breach. Lersundi et al. [15] and Bhamra et al. [16] similarly support combined approaches, reporting 10% and 9% recurrence rates, respectively.

We proceeded to distal phalangectomy based on three converging considerations: oncologic (confirmed cortical breach rendering adequate intralesional margins unachievable), anatomic (the second toe distal phalanx is functionally expendable), and patient-centered (progressive cosmetic deformity and recurrence-related anxiety that, in the setting of a benign tumor, reasonably weighed in favor of definitive intervention [17,18,19]). The patient-centered dimension of this decision—an adolescent’s appearance-related and psychological concerns—is rarely acknowledged in the CMF literature, where operative indications have been framed almost exclusively in oncologic terms. In a malignant tumor, those concerns do not change the operation; in a benign recurrent one, they can and should inform the threshold for intervention. Complete excision achieved reliable local tumor control without significant functional compromise.

In summary, CMF in a small tubular bone can recur years after curettage with bone grafting—these patients warrant long-term radiographic surveillance [5,13]. When recurrence does occur, the decision about what to do next is not purely oncologic: in a benign tumor, a patient’s cosmetic and psychological concerns are legitimate surgical considerations. Several limitations apply: intraoperative photographs were not obtained at the initial surgery; no formal anxiety or cosmetic scoring instrument was administered, as the surgical indication was not symptom-driven and standardized tools were not applied prospectively; and GRM1 immunohistochemistry was not performed, as it was not yet in routine clinical use at the time of diagnosis. Future reports should incorporate validated instruments—such as Patient-Reported Outcomes Measurement Information System (PROMIS^®^) Anxiety for psychological burden—alongside functional performance measures. A multidisciplinary approach incorporating clinical, radiologic, and pathologic correlation remains essential to avoid overdiagnosis and overtreatment in this rare entity.

## 4. Conclusions

**Figure 5:**
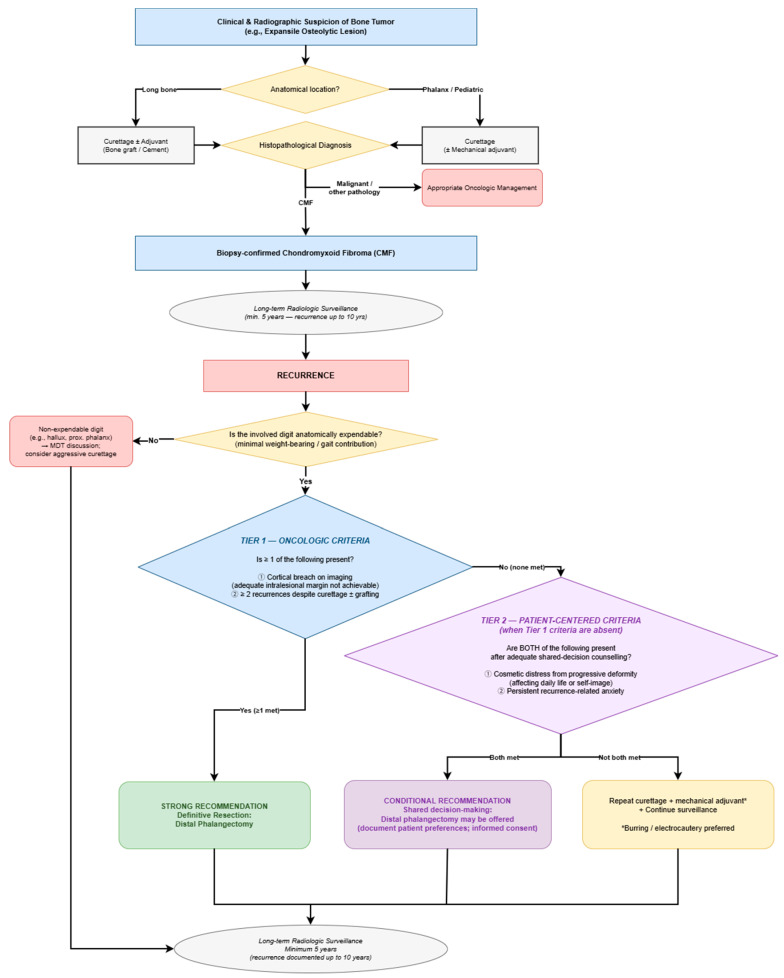
Proposed diagnostic and therapeutic algorithm for chondromyxoid fibroma [2,4,5,6,13,17,18].

CMF of the toe phalanx may show delayed recurrence after curettage, requiring prolonged radiologic surveillance. For primary lesions, digit-preserving curettage remains appropriate. In recurrent cases, definitive resection should be considered based on a convergence of oncologic, anatomic, and patient-centered factors; distal phalangectomy may provide reliable local control in anatomically expendable digits, though longer follow-up is warranted to confirm durability (Figure 5).

## Figures and Tables

**Table 1:** Reported cases of chondromyxoid fibroma involving toes or fingers.

Author/Year	Location	Age	Treatment	Recurrence
Present case	Second toe distal phalanx	10 years	Curettage → distal phalangectomy	Yes (4 years)
Jilani et al., 2024 [9]	Great toe distal phalanx	7 years	Curettage + bone graft	No (6 months)
Khan et al., 2025 [7]	5th finger middle phalanx	47 years	Local excision	No (9 months)
Hwang et al., 2014 [10]	2nd finger middle phalanx	35 years	Aggressive curettage + bone graft	No (15 months)
Kashyap et al., 2023 [14]	Great toe distal phalanx	35 years	Curettage	No (6 months)
Vasudeva et al., 2020 [8]	Great toe distal phalanx	11 years	Curettage → re-curretage + bone graft	Yes (6 months)
Mallya et al., 2018 [13]	Great toe (1st MT to distal phalanx)	24 years	Curettage + bone graft → amputation	Yes (6 years)

## Data Availability

The data presented in this study are available on request from the corresponding author. The data are not publicly available due to privacy and ethical reasons.

## Abbreviations

The following abbreviations are used in this manuscript:

CMF	Chondromyxoid fibroma
DBM	Demineralized bone matrix
SMA	Smooth Muscle Actin
PMMA	Polymethylmethacrylate
PROMIS^®^	Patient-Reported Outcomes Measurement Information System
